# Targeting a Tumor-Specific Epitope on Podocalyxin Increases Survival in Human Tumor Preclinical Models

**DOI:** 10.3389/fonc.2022.856424

**Published:** 2022-05-04

**Authors:** Diana Canals Hernaez, Michael R. Hughes, Yicong Li, Ilaria Mainero Rocca, Pamela Dean, Julyanne Brassard, Erin M. Bell, Ismael Samudio, Anne-Marie Mes-Masson, Yoshiki Narimatsu, Henrik Clausen, Ola Blixt, Calvin D. Roskelley, Kelly M. McNagny

**Affiliations:** ^1^ The Biomedical Research Centre and School of Biomedical Engineering, University of British Columbia, Vancouver, BC, Canada; ^2^ Department of Biotechnology and Biomedicine, Technical University of Denmark, Lyngby, Denmark; ^3^ Department of Cellular and Physiological Sciences, University of British Columbia, Vancouver, BC, Canada; ^4^ Centre for Drug Research and Development, Vancouver, BC, Canada; ^5^ Department of Medicine,Institut du Cancer de Montreal, Montreal, QC, Canada; ^6^ Copenhagen Center for Glycomics, Department of Cellular and Molecular Medicine (ICMM), University of Copenhagen, Copenhagen, Denmark

**Keywords:** podocalyxin, antibody-drug conjugate, PODO447, tumor-specific, glycoepitope

## Abstract

Podocalyxin (Podxl) is a CD34-related cell surface sialomucin that is normally highly expressed by adult vascular endothelia and kidney podocytes where it plays a key role in blocking adhesion. Importantly, it is also frequently upregulated on a wide array of human tumors and its expression often correlates with poor prognosis. We previously showed that, in xenograft studies, Podxl plays a key role in metastatic disease by making tumor initiating cells more mobile and invasive. Recently, we developed a novel antibody, PODO447, which shows exquisite specificity for a tumor-restricted glycoform of Podxl but does not react with Podxl expressed by normal adult tissue. Here we utilized an array of glycosylation defective cell lines to further define the PODO447 reactive epitope and reveal it as an O-linked core 1 glycan presented in the context of the Podxl peptide backbone. Further, we show that when coupled to monomethyl auristatin E (MMAE) toxic payload, PODO447 functions as a highly specific and effective antibody drug conjugate (ADC) in killing ovarian, pancreatic, glioblastoma and leukemia cell lines *in vitro*. Finally, we demonstrate PODO447-ADCs are highly effective in targeting human pancreatic and ovarian tumors in xenografted NSG and Nude mouse models. These data reveal PODO447-ADCs as exquisitely tumor-specific and highly efficacious immunotherapeutic reagents for the targeting of human tumors. Thus, PODO447 exhibits the appropriate characteristics for further development as a targeted clinical immunotherapy.

## Introduction

Despite dramatic improvements in early detection, diagnosis and treatment, cancer remains one of the leading causes of death worldwide with an estimated 10 million deaths in 2020 (1). Importantly, over 90% of these cancer-related deaths are the result of metastatic disease which remains the most difficult to treat. This is largely due to its diffuse nature and the paucity of effective immuno- and chemotherapeutic agents with the appropriate selectivity for targeting tumor cells while sparing normal healthy tissue (2).

Podocalyxin (Podxl), a member of the CD34-family of sialomucins (3), is normally expressed by embryonic (ES) and induced pluripotent (iPS) stem cells, early hematopoietic progenitor, adult vascular endothelia and kidney podocytes (4–11). Podxl plays an essential role in murine and human development and null mutations lead to perinatal lethality (4, 12, 13). Functionally, we and others have shown that this molecule is targeted to the apical domain of cells where it plays a key role in blocking adhesion, opening luminal structures and enhancing cell mobility and invasiveness (8, 14).

Importantly, in addition to its normal tissue expression pattern, Podxl is also aberrantly expressed by a wide variety of human tumors and its expression is consistently an indicator of poor prognosis (10, 15–25). Recent gene silencing and xenograft studies reveal an essential role for Podxl in disease progression through enhancing the mobility and invasiveness of tumor cells and promoting formation of distal metastasis (16, 26–28). Accordingly, Podxl has garnered increasing attention as a target for immunotherapy and we previously identified a core protein binding antibody (PODO83/PODOC1) that slows primary tumor growth and blocks metastatic disease (16). Nevertheless, further development of Podxl core protein-binding antibodies as cancer immunotherapeutics has been hampered by concerns over possible toxicity to normal vascular and renal tissue where Podxl is abundantly expressed in healthy adults.

The 53 kDa Podxl core protein undergoes extensive, tissue-specific N- and O-linked glycosylation leading to a mature protein with an apparent molecular weight of 150-200 kDa (8, 10, 14). Since glycosylation processes are frequently altered in cancer (29–31), we recently engaged in an antibody development campaign to identify antibodies that would recognize tumor specific glycoepitopes on Podxl but not react with Podxl expressed by normal tissue (32). One resultant antibody, PODO447, exhibits exquisite specificity for a tumor glycoform of Podxl but lacks reactivity with normal adult human tissue. Furthermore, in a tissue microarray (TMA) screen we showed that this epitope is expressed by over 60% of high grade serous (HGS) ovarian tumors (32). Thus, this antibody exhibits the appropriate specificity and distribution for further development as a therapeutic, particularly for highly metastatic disease.

In the current work, we evaluate the ability of PODO447 to serve as the targeting arm for therapeutic drug delivery in ADC assays. We find that although this antibody lacks inherent Podxl function blocking activity, it is highly effective in killing a variety of tumor cells *in vitro* as an ADC. Moreover, we find that PODO447-ADC shows potent efficacy against human tumor cell lines in two different mouse xenograft models. Finally, using a cell-based glycan array we reveal the PODO447-reactive epitope to be a core 1 O-glycan in the context of the Podxl polypeptide. In aggregate, these data reveal PODO447-ADC as a highly specific and efficacious immunotherapeutic agent and highlight its promise for treating high grade human tumors.

## Materials and Methods

### Cell Culture

HEK-293 WT, HUVEC, SKOV3, A-172, MIA PaCa-2, THP-1 and MDA-MB-231 cells were obtained from the American Tissue Culture Collection (ATCC). Glycosylation-mutant HEK-293 cells (33, 34) and patient tumor-derived epithelial high grade serous ovarian OV3331 and TOV3133D cancer cell lines (35, 36) were previously described. ONS-76 medulloblastoma cell line was generously provided by Dr. Sorensen from the University of British Columbia. PODO447-positive and PODO447-negative CFPAC-1 cells were generated by sorting parental pancreatic CFPAC-1 cells (metastatic tumor-derived) with or without PODO447 expression. HEK-293 WT and isogenic cells were grown in DMEM (Gibco, #11965-092) supplemented with 10% FBS and 2mM GlutaMAX (Gibco, #35050061). CFPAC-1 cells were grown in IMDM (Gibco, #12440053) supplemented with 10% FBS. MIA PaCa-2, A-172, and MDA-MB-231 cells were grown in DMEM (Gibco, #11965-092) supplemented with 10% FBS and 10 U/ml penicillin and streptomycin (P/S) (Gibco, #15140-122). Human umbilical vein endothelial (HUVEC) cells were harvested from donor umbilical cords (Human Ethics no. H10-00643), grown in Endothelial Cell Growth Medium-2 Bulletkit™ (LONZA, #CC-3162), and used between passages 2 and 8. Human ovarian cancer SKOV3 cells were grown in DMEM F-12 with 15 mM HEPES (Sigma, #D6421) supplemented with 10% FBS, 0.2 mM L-glutamine (Gibco, #25030-081) and 10 U/ml P/S. OV3331 and TOV3133D cells were grown in complete OSE medium (36). ONS-76 cells were grown in RPMI (Gibco, #11875093) supplemented with 10% FBS. All cell lines were maintained at 37°C, 5% CO_2_ and high humidity.

### Flow Cytometry

Cells were washed 1X with Ca^2+^- and Mg^2+^-free HBSS (Gibco, #14170-112), incubated for 1-2 min at 37°C in a 0.25% trypsin solution, quenched with complete growth media, then centrifuged for 4 min at 394*g*, washed 2X with FACS buffer (PBS, 2 mM EDTA, 5% FBS, 0.05% sodium azide) and transferred to a 96 well ‘v’ bottom plate. Cells were resuspended in 100 μl blocking buffer (FACS buffer, 1 μg/ml of anti-CD16/CD32 (clone 2.4G), 2% rat serum) for 20 min at 4°C in the dark, then spun at 394*g* for 4 min and incubated in 100 μl primary antibody (Ab) solution for 30 min at 4°C in the dark. Rabbit-PODO83 (16) (2 μg/ml); and either rabbit or chimeric PODO447 (32) (5 μg/ml) were used to detect Podxl. Biotinylated pan-lectenz lectin (1 μg/ml, Lectenz-Bio #SK0501B) was used to detect sialylated complex glycans. 3C9 (37), 5F4 (38) and TKH2 (39) antibodies were used to detect core 1, Tn and STn glycostructures, respectively. Wisteria Floribunda lectin (WFA, 0.25 μg/ml, Vector Laboratories, #B-1355-2) was used to detect α/β N-acetylgalactosamine (α/β-GalNAc). Erythrina Cristagalli lectin (ECL, 1 μg/ml, Vector Laboratories, #B-1145-5) was used to detect galactose, lactose and N-acetylgalactosamine. Peanut agglutinin (PNA, 5μg/ml, Vector Laboratories #B1075-5; ThermoFisher #L32459) was used to detect T-antigen. Rabbit-IgG (5 μg/ml, Vector Laboratories, #I-1000-5) and mouse/human-chimeric palivizumab (5 μg/ml, National Research Council, NRC) were used as isotype controls. Next, cells were washed 3X with FACS buffer and resuspended in 100 μl of secondary Ab solution (Alexa Fluor 647 (AF647) donkey-anti-rabbit (2 μg/ml, Invitrogen, #A31573); AF647 goat-anti-human (2 μg/ml, Jackson ImmunoResearch Laboratories, #109-605-098); Brilliant Violet 450 (BV450) Streptavidin (2 μg/ml)) for 30 min at 4°C in the dark. Cells were washed 2X with FACS buffer and resuspended in FACS buffer containing propidium iodide (PI) (0.5 μg/ml, Life Technologies, #P3566). High-throughput flow cytometry data was acquired using an SA3800 spectral analyzer. The remaining flow cytometry data was acquired using a BD LSRII and analyzed using FlowJo™ software (BD Biosciences, Ashland).

### *In Vitro* Antibody Internalization

Sub-confluent cells were harvested and plated in 50 μl per well at the following concentrations: MIA PaCa-2 (8 x 10^3^ cells/well), A-172 (8 x 10^3^ cells/well) and SKOV3 (1 x 10^4^ cells/well). Cells were then incubated overnight at 37°C, in 5% CO_2_. On day 1, coupling of the antibodies was performed by incubation of biotinylated PODO447 or palivizumab control mAb (40) with pHrodo™ Red Avidin (ThermoFisher, #P35362) at 1:1 molar ratio for 30 min at 4°C. Next, 50 μl of cold pHrodo™-mAb mix was added to the cells and the plate was placed in the Incucyte®. The Incucyte® ZOOM software was set in “standard” mode with the phase and red channels selected and set to scan every 10 min for 24 hours. The rate of antibody internalization was evaluated through increased fluorescent area, reported as total internalization area (μm^2^/well). Average internalization was calculated, and statistical analyses was performed using two-way ANOVA in GraphPad Prism software.

### *In Vitro* Antibody Cytotoxicity

Cells were plated on 96-well plates in 100μl culture medium and allowed to adhere overnight. SKOV3, MIA PaCa-2, A-172, THP-1 and HUVEC were seeded at 2.5 x 10^3^ cells per well; OV3331, TOV3133D and ONS-76 were seeded at 5 x 10^3^ cells per well. On day 1, a 5X stock solution of each mAb/ADC concentration to be tested was prepared in a stepwise 1:3 serial dilution series in cell culture medium, and 25μl of each dilution was added to cells in triplicate. Treated cells were cultured at 37°C, 5% CO_2_ and high humidity for 144 h. On day 6, thiazolyl blue tetrazolium bromide (MTT) assays were used to determine relative cytotoxicity. Briefly, 100μl of MTT (1mg/ml, Thermo Fisher Scientific, #AC158990010) was added to the cells and left to incubate for 3 h at 37°C. Next, MTT media was removed and 50μl of DMSO was added to the cells and allowed to incubate for 15 min at RT, protected from light and with mild shaking. Absorbance was then read at 570nm. Percent viability was calculated as [1-(absorbance of treated samples/average absorbance of control samples)] x 100. Average relative cytotoxicity was calculated, and statistical analyses were performed using two-way ANOVA (GraphPad Prism software).

### Antibody-Dependent Cell-Mediated Cytotoxicity Assay

A-172 (2x10^6^) cells were stained with 10μM CellTrace™ Far Red (Invitrogen™, #C34572) for 30 min at 37°C. Next, cells were washed 2X and seeded at 2 x 10^3^ cells per well on a 96-well plate and allowed to adhere. 16 h after seeding A-172 labelled cells were incubated with 2.5, 0.5, or 0.1 μg/ml of PODO447, and 2.5 μg/ml of control human IgG1 for 15 min at RT. Next, antibody-labelled A-172 cells were co-cultured with human PBMCs at a 7:1 and 3:1 ratio for 1 h at 37°C, 5% CO_2._ Cells were then harvested and stained with 1 μg/ml of PI for 5 min at RT. Cell viability was assessed *via* flow cytometry using a BD LSRFortessa™ X-20 and analyzed using FlowJo™ software (BD Biosciences, Ashland).

### ADC Linker Stability Assay

Human cord blood CD34+ cells (1x10^6^ cells/well, STEMCELL Technologies, # 70008.2) were seeded in 200 µL of complete growth medium [X-Vivo™ 15 (Lonza, #BE02-060F), rhIL-3 (20 ng/mL, Peprotech, #200-03), rhIL-6 (20 ng/mL, Peprotech, #200-06), rhSCF (100 ng/mL, Peprotech, #AF-300-07) and rhFlt-3L (100 ng/mL, Peprotech, #300-19)] in round-bottom 96-well plates and cultured at 37°C in 5% CO_2_. On day 3, cells were washed 2X with X-Vivo™ 15 medium and incubated in X-Vivo™ 15 medium supplemented with rhSCF (50 ng/mL), rhFlt-3L (100 ng/mL), rh-IL3 (5 ng/mL), rhGM-CSF (5 ng/mL, Peprotech, #300-03) rhG-CSF (5 ng/mL, Peprotech, #300-23) at 37°C in 5% CO_2_ for 4 days. On day 7, cells were then washed 2X and incubated in X-Vivo™ 15 medium supplemented with rhIL-3 (5 ng/mL) and rhG-CSF (30 ng/mL) for an additional 4 days. On day 11, cells were washed 2X and cultured in X-Vivo™ 15 medium supplemented with rhG-CSF (30 ng/mL) and either PODO447- or palivizumab-Vedotin at concentrations ranging from 0.01 – 10 µg/mL for an additional 6 days. On day 17, cells were harvested and prepared for flow cytometric analysis, as follows. Cells were blocked with 10% FBS and 20 µg/mL human IgG at 4°C for 10 minutes. Following blocking, cells were stained with anti-CD66b antibody (Biolegend, #305106) and DAPI (Biolegend, #422801) at 4°C for 20 minutes. Cells were then washed 2X and resuspended in FACS buffer. Flow cytometry data was acquired using a Beckman Coulter CytoFlex and analyzed using FlowJo™ software (BD Biosciences, Ashland) This assay was performed by ReachBio Research Labs, Seattle, USA (www.reachbio.com). The effect of ADCs on neutrophil differentiation is represented by percentage of CD66b+ cells within the viable cell population.

### Mice

Tumor model animal experiments were carried out using 6-12-week-old female NOD.Cg-*Prkdc* ^scid^ *Il2rg^t^* ^m1Wjl^/SzJ (NSG) and NU/J (nude) mice obtained from Jackson’s Laboratories (#005557 and #002019, respectively). Animals were bred in a specific, pathogen-free facility and all experiments were carried out under approved University of British Columbia Animal Care committee protocols (ACC protocol #A16-0007 and A20-0042) based on the recommendations of the Canadian Council on Animal Care.

### Pre-Clinical *In Vivo* Xenograft Models to Assess PODO447-ADC Efficacy

MIA PaCa-2 (1x10^6^) or OV3331 (1x10^6^) cells were injected subcutaneously into the right flank of NSG or nude mice. Tumor dimensions were measured twice a week and tumor volumes (cm^3^) were calculated by [(length x width^2^)/2]. Once tumors reached 0.15cm^3^, mice were treated with either PODO447- or palivizumab-Vedotin at concentrations ranging from 4 – 2 mg/kg. ADC treatments were administered intravenously every 4 days. Average tumor volume over time was calculated, and statistical analysis was performed using two-way ANOVA test in GraphPad Prism software. Endpoints for survival analyses included animals with tumors exceeding 1 cm^3^ or any animals with signs of morbidity reaching a humane endpoint regardless of tumor size. Survival Kaplan-Meier curves were calculated, and statistical analysis was performed using a Log-rank (Mantel-Cox) test in GraphPad Prism software.

### Immunohistochemistry

Tumor slides were stained with either rabbit-PODO83 (5 μg/ml), Rbt/Hu-chimeric PODO447 (1 μg/ml), Mo/Hu-chimeric pavilizumab control (1 μg/ml) or rabbit IgG control (5 μg/ml, Vector laboratories, #I-1000-5). Briefly, slides were deparaffinized and rehydrated in 100% xylene (3X, 5 min), 100% ethanol (2X, 3 min), 95% ethanol (1X, 3 min), 70% ethanol (1X, 3 min) and distilled water (1X, 3 min). Antigen retrieval was performed by heating slides in citrate buffer at 90°C for 40 min. Slides were washed in PBS (3X, 5min), incubated for 30 min in blocking solution (PBS, 5% donkey serum, 0.5% BSA, 0.3% Triton X-100), and incubated in primary Ab solution overnight at 4°C. Slides were then washed with TBST (3X, 15 min), incubated with either anti-rabbit-BIOT (2 μg/ml, SouthernBiotech, #6440-06) or anti-human-BIOT (2 μg/ml, Jackson ImmunoResearch, #209-065-098) in blocking solution for 30 min at RT, and washed again with TBST (4X, 15 min). Endogenous peroxidase was blocked with 3% hydrogen peroxide in methanol for 40 min at RT prior to rinsing 3X with PBS. Signal was amplified using Vectastain® Elite® ABC HRP Kit (Vector Laboratories, #PK-6100) according to manufacturer’s instructions, and then washed with PBS (3X, 5min). Signal was visualized using a DAB peroxidase (HRP) Substrate Kit (Vector Laboratories, #SK-4100) following the manufacturer’s recommendations. Each slide was incubated with DAB solution for 30-40 sec until strong signal appeared, then the reaction was stopped by dilution with PBS. Slides were counterstained with Harris Hematoxylin (2.5 min, Fisher HealthCare™ PROTOCOL™, #23-245651), rinsed 1X with distilled water (30 sec), incubated with differentiator solution (30 sec, 1% HCl in 70% EtOH) and rinsed with distilled water (30 sec). Staining was fixed in Bluing Solution (0.1% sodium bicarbonate) for 30 sec and rinsed in distilled water (1X, 30 sec) prior to dehydration in graded alcohols and xylene. Slides were mounted using Permount solution (Fisher Scientific, #SP15100) and subsequently analyzed for Podxl staining.

## Results

### PODO447 Binds to a Core 1 Glycostructure on Podxl

**Figure 1:**
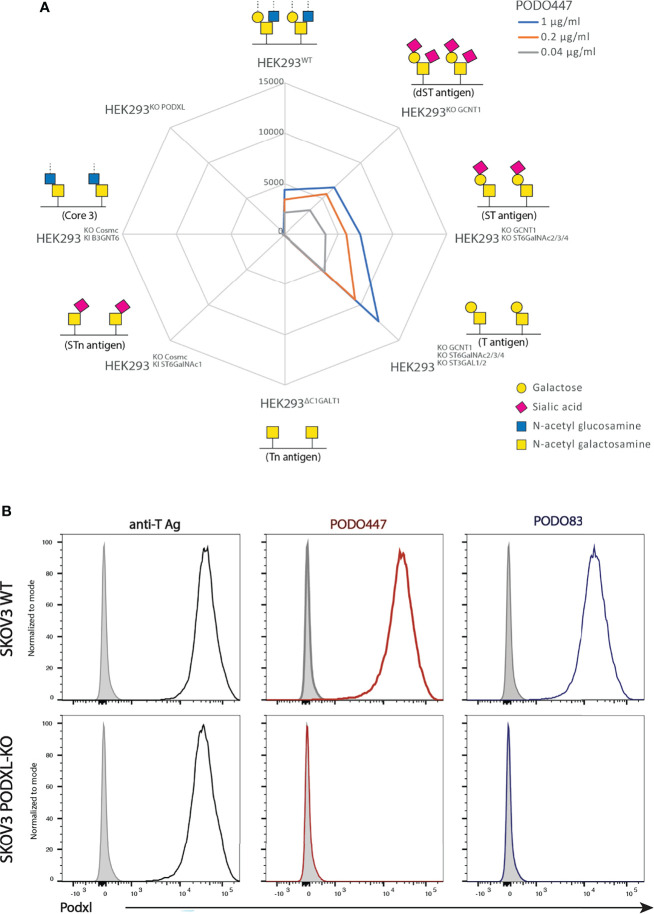
PODO447 binds to a core 1 O-glycan structure on Podxl. **(A)** Isogenic HEK-293 cells bearing mutations in glycosyl transferases were stained with various concentrations of PODO447 and evaluated by flow cytometry. Mean Fluorescence Intensity (MFI) results are summarized in the “spiderweb plot”. **(B)** Flow cytometric anti-core 1 PNA lectin (black lines), PODO447 (red lines) and PODO83 (blue lines) binding profiles of SKOV3 WT and SKOV3 PODXL-KO cells.

Previously we showed that PODO447 exhibits high affinity for tumor-specific glycoepitopes using a well-characterized printed glycan array (32, 41). One limitation of these arrays, however, is that they present glycostructures in isolation and not in the context of their normal attachment to proteins or lipids (41). Thus, while they provide guidance on the types of carbohydrate structures antibodies detect, they lack the full antigenic context for determining their true specificity. Accordingly, to gain further insight into PODO447’s specificity in a natural context, we probed its reactivity against a cell-based glycan array composed of isogenic HEK-293 cells, which express endogenous Podxl, with a combination of knockout (KO) and knock-in (KI) glycosyltransferase genes that control the loss or gain of core 1 and core 2 O-glycosylation (33). First, we verified that the glycoengineered HEK-293 cells correctly displayed the predicted O-glycoforms using a variety of lectins and monoclonal antibodies (mAbs) (**Supplementary Figure 1**). Next, we probed this cell array with PODO447. In contrast to the printed array, PODO447 failed to recognize LacDiNAc glycostructures on HEK-293 cells and, instead, exhibited selective binding for core 1 O-glycans as demonstrated by the lack of Ab recognition after the loss of core 1 structures (Tn, KO C1GALT1), and increased Ab binding after the loss of core 2 O-glycans (T/ST/dST, KO GCNT1) (**Figure 1A**). Additionally, loss of α6 (KO ST6GALNACT2/3/4) and α3 (KO ST3GAL1/2) sialylation increased Ab binding suggesting that sialylation, although tolerated, is dispensable for PODO447 binding. Lastly, we also tested PODO447 recognition of cancer-associated STn (KI ST6GALNAC1/KO COSMC) and core 3 O-glycans (KI B3GNT3/KO COSMC) and observed no Ab binding to either of these structures. From these data, we conclude that PODO447 binds preferentially to a non-sialylated core 1 O-glycan, also known as T-antigen (T), a glycomotif that has been previously reported as a cancer-associated glycan epitope (42). Importantly, PODO447 recognizes T-antigen only in the context of the Podxl backbone since PODO447 fails to bind to PODXL-KO HEK-293 cells (**Figure 1A**).

To further validate the contribution of Podxl’s peptide backbone to the PODO447 epitope, we evaluated the binding of PODO447, PODO83 and peanut agglutinin (PNA) to SKOV3 wild type (WT) and Podxl knock-out (PODXL-KO) cells. The Podxl core protein-binding Ab, PODO83 (32), served as a control for ablation of protein expression, while PNA served as a control for T-antigen expression by all cell surface proteins. We found that PNA bound both SKOV3 WT and SKOV3 PODXL-KO cells suggesting a wide array of T-antigen epitopes are expressed on these cell lines (**Figure 1B**). Conversely, PODO447 and PODO83 Abs reacted exclusively with WT but not PODXL-KO cells, confirming the specificity of these Abs for epitopes linked to the Podxl core protein. In addition, we further validated the contribution of the T-antigen to the PODO447 epitope using the human pancreatic CFPAC-1 cell line. This cell line shows heterogeneous expression of the PODO447 epitope (low to high) and a linear correlation between PODO447 and PNA staining. This was further validated by sorting this line into PODO447-positive and -negative CFPAC-1 cell subclones which, correspondingly, showed high and low PNA staining, respectively (**Supplementary Figure 2**). In summary, these experiments suggest that PODO447 specifically binds a glyco-peptide epitope consisting of the core 1 O-glycan in the context of the Podxl polypeptide but does not recognize general core 1 glycans decorating other proteins.

### PODO447 Lacks a Direct Effect on Cell Viability

**Figure 2:**
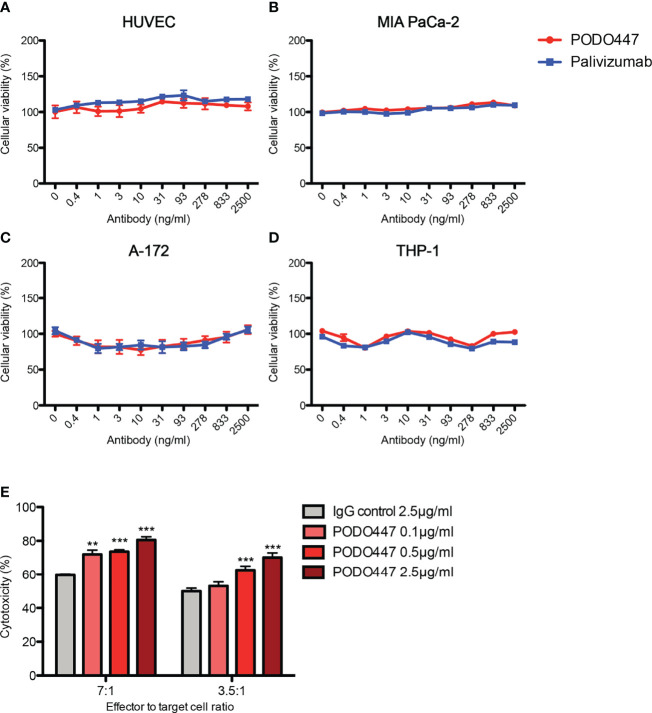
Unconjugated PODO447 lacks inherent cytotoxic activity but induces ADCC. Cytotoxic effect of unconjugated PODO447 and palivizumab control Abs on **(A)** human umbilical vein endothelial cells (HUVEC), **(B)** pancreatic ductal adenocarcinoma (MIA PaCa-2), **(C)** glioblastoma (A-172) and **(D)** acute monocytic leukemia (THP-1) cell lines as determined by MTT assay; and **(E)** antibody-dependent cellular cytotoxicity induced by PODO447 in A-172 cells after 1 hr co-incubation with human PBMCs at a 7:1 and 3.5 ratio of effector (PBMCs) to target (A-172) cells. ADCC-induced cellular toxicity was determined by flow cytometry. **p < 0.01, ***p < 0.001.

Prompted by previous reports of anti-Podxl Abs with cytotoxic activity against undifferentiated human embryonic stem cells (43), and our own data showing PODO83 blocks metastasis *in vivo* (16), we investigated whether PODO447 binding exhibits any effects on cell viability. We performed a long-term (144h) *in vitro* exposure assay using unconjugated PODO447 or palivizumab, an Ab specific for the F protein of the respiratory syncytial virus (RSV) that served as a control. We used normal human endothelia (HUVEC) cells and an array of human cancer cell lines, including pancreatic (MIA PaCa-2), glioblastoma (A-172) and acute monocytic leukemia (THP-1, AML) lines (**Figures 2A** **–D**). Unconjugated PODO447 had no effect on cell viability at any concentration, suggesting that binding of this antibody does not block pathways critical for cancer cell survival and proliferation *in vitro*.

### PODO447 Induces Cellular Toxicity *Via* ADCC

We next evaluated if binding of PODO447 induces antibody-dependent cell-mediated cytotoxicity (ADCC). We first incubated A-172 cells with 0.1, 0.5 and 2.5 μg/ml of PODO447 or 2.5 μg/ml of palivizumab control antibody. Next, we co-cultured the antibody-bound cells with human peripheral blood mononuclear cells (PBMCs) at 7:1 and 3.5:1 ratio of effector (PBMCs) to target (A-172) cells and assessed A-172 cell viability by flow cytometry. We found that PODO447 induced a modest, dose-dependent, level of ADCC activity on A-172 cells at both effector to target cell ratios (**Figure 2E**). From these results we conclude that unconjugated PODO447 can affect tumor cell viability *via* ADCC.

### Evaluation of PODO447 Antibody-Drug Conjugates

**Figure 3:**
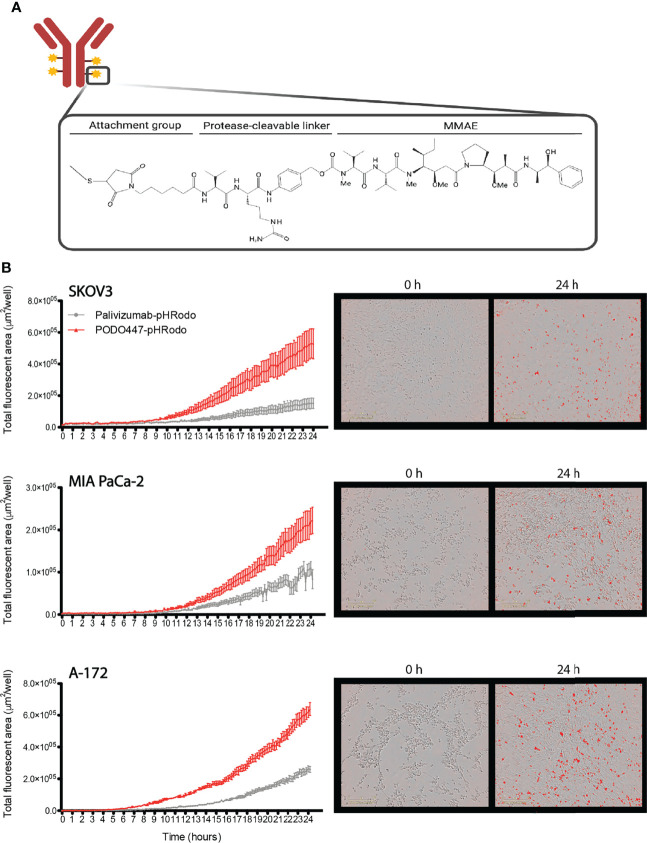
PODO447 internalization on cancer cells. **(A)** Representative image of mAb-Vedotin structure (created with Biorender.com). **(B)** Internalization of PODO447- and palivizumab-pHRodo conjugates on SKOV3 (*P* < 0.001), MIA PaCa-2 (*P* < 0.001), and A-172 (*P* < 0.001) cells. Left graphs show internalization over 24h as determined by Incucyte® evaluation of the increase of total fluorescent area per well. Pictures on the right are representative images of PODO447 internalization at 0- and 24-h timepoints.

Since unconjugated PODO447 did not directly affect cancer cell survival and only showed a low level of ADCC activity, we next investigated the potential use of PODO447 as the targeting arm for an ADC. We selected monomethyl auristatin E (MMAE) as the toxic payload since it is a well-studied tubulin polymerization inhibitor that, when internalized into cells, exhibits potent and broad anti-tumor activity (44). MMAE was conjugated to either PODO447 or palivizumab control. Coupling was accomplished using a valine-citrulline (val-cit) proteolytically cleavable linker, resulting in “Vedotin” (Val-Cit-PABC-MMAE)-ADCs (**Figure 3A**). Linker stability was independently assessed by ReachBio Research Labs (RRL, www.reachbio.com) using an *in vitro* neutrophil differentiation killing assay, where extracellular proteolysis of the cleavable linker results in neutrophil cytotoxicity (45). RRL found that PODO447-ADC behaved very similarly to palivizumab control-ADC in the presence of neutrophils (**Supplementary Figure 3A**), thus suggesting both antibodies induce similar low levels of off-target toxicity as a result of non-specific extracellular cleavage.

Since these ADCs are dependent on internalization into acid compartments for cleavage and toxicity, we first tracked the ability of membrane-bound PODO447 to be internalized by tumor cells. Biotinylated PODO447 or palivizumab control Ab were conjugated to pHrodo™ Red Avidin (Invitrogen™), a pH sensitive dye that becomes fluorescent in acidic intracellular environments. Importantly, neither MMAE nor biotin conjugation altered the binding profile of PODO447 (**Supplementary Figures 3B, C** **)**. We monitored antibody internalization by SKOV3, A-172 and MIA PaCa-2 cultures over 24 hours (**Figure 3B**) and found that PODO447 was robustly internalized by all cell lines tested. In contrast, palivizumab control Ab exhibited negligible background internalization. Thus, PODO447-ADC is competent to deliver a cytotoxic payload to the intracellular compartment of Podxl-expressing tumor cells (**Figure 3B**).

**Figure 4:**
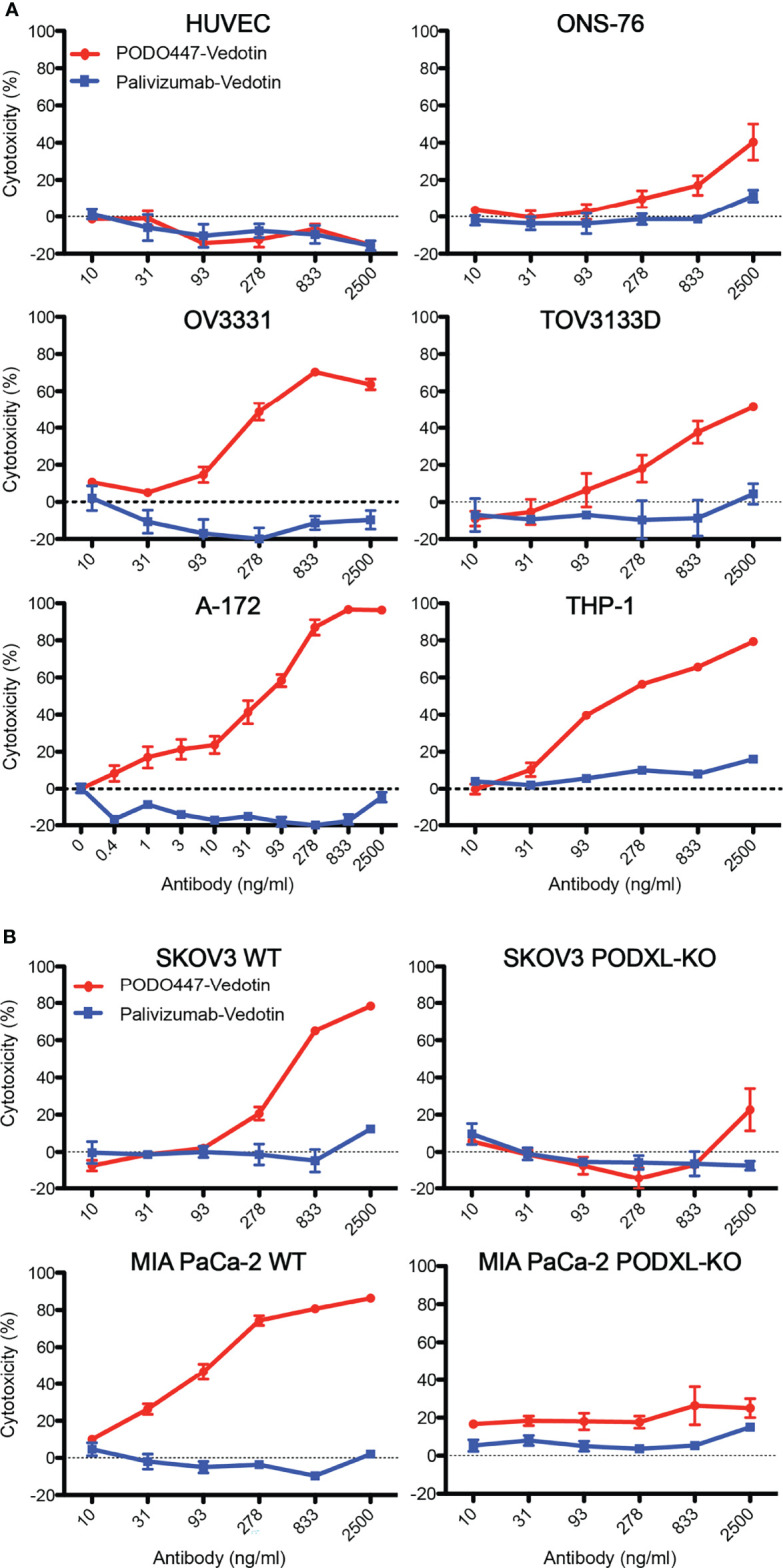
*In vitro* cytotoxicity and selectivity of PODO447-Vedotin. Cytotoxic effect of PODO447- and palivizumab-Vedotin conjugates on **(A)** HUVEC (ns), ONS-76 (*P* < 0.001), OV3331 (*P* < 0.001), TOV3133D (*P* < 0.001), A-172 (*P* < 0.001), THP-1 (*P* < 0.001) and **(B)** SKOV3 (*P* < 0.001) and MIA PaCa-2 (*P* < 0.001) WT and PODXL-KO (ns) cells. Cells were assessed for viability by MTT assay after 144 h of continuous exposure. Horizontal line at 0% cytotoxicity is representative of untreated parental growth rate.

To evaluate PODO447-ADC cytotoxic activity, we selected a panel of Podxl-expressing human cancer cell lines, including SKOV3 (ovarian, ascites-derived), MIA PaCa-2 (pancreatic, primary tumor-derived), A-172 (glioblastoma, primary tumor-derived), ONS-76 (medulloblastoma, primary tumor-derived), THP-1 (acute monocytic leukemia), and patient tumor-derived TOV3133D and OV3331 [ovarian, primary solid tumor- and ascites-derived, respectively (35, 36)] (**Figure 4A**; **Supplementary Figure 4**). Cells were treated with serial dilutions of PODO447- or palivizumab-ADC for 6 days and then evaluated for cell viability. Although we saw a significant but modest toxicity (~40%) on ONS-76 cells at 2500 ng/ml, we observed potent cytotoxicity against THP-1 and A-172 cells with an IC_50_ of 31 ng/ml and 93 ng/ml, respectively, and over 90% cytotoxicity at 278 ng/ml and higher PODO447-Vedotin doses. While the cytotoxic effect of PODO447-ADC on TOV3133D cells was modest (IC_50_ of 833 ng/ml), we observed a potent cytotoxic effect in OV3331 cells with an IC_50_ of 278 ng/ml and 80% cytotoxicity at 833 ng/ml. In contrast, no significant toxicity was observed against any cell lines treated with control palivizumab-ADC. Importantly, PODO447-ADC treatment of HUVEC cells, which express the Podxl core protein but not the PODO447 epitope, led to no significant toxicity. In summary, these data suggest that PODO447-ADC exhibits specific toxicity against a broad array of Podxl-expressing tumor cell lines and is well tolerated by cells expressing the native Podxl polypeptide but lacking the tumor-specific PODO447 glycoepitope.

To further confirm that PODO447-ADC cytotoxicity is linked to binding of the PODO447 epitope in the context of the Podxl core protein, we assessed *in vitro* killing of WT and PODXL-KO SKOV3 and MIA PaCa-2 cell lines (32) (**Figure 4B**). PODO447-ADC led to a 3-fold reduction in the viability of SKOV3 WT cells and an 8-fold reduction in the viability of MIA PaCa-2 WT cells compared to control ADC-treated cells. In both cell types, this cytotoxic effect was highly specific as we did not observe a reduction in cell viability of palivizumab-ADC treated cells, or in PODXL-KO control cells treated with PODO447-ADC (**Figure 4B**). We conclude that PODO447 is effective in delivering toxic payloads to tumor cells expressing an appropriately glycosylated form of Podxl.

### PODO447-ADC Leads to Regression of Xenografted Human Ovarian and Pancreatic Tumors

**Figure 5:**
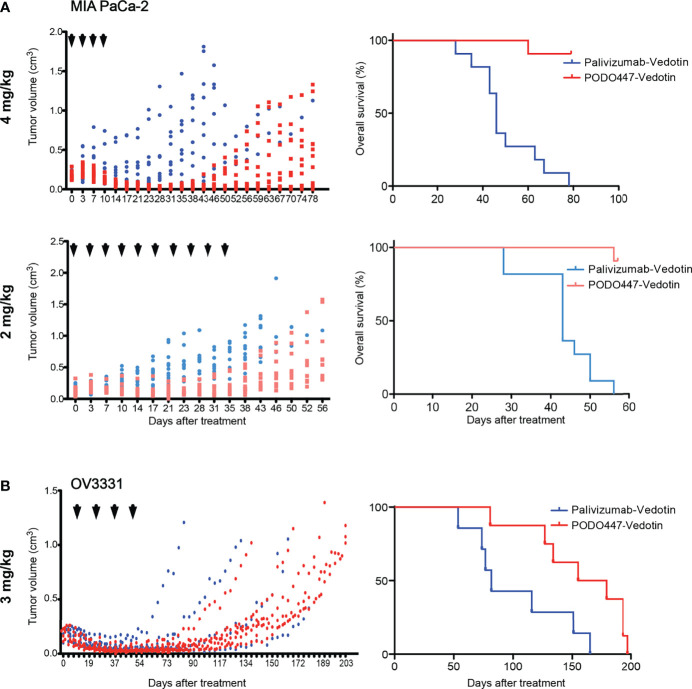
Therapeutic effect of PODO447 on pancreatic and ovarian tumor-bearing NSG mice. Assessment of PODO447- (blue) and palivizumab-ADC (red) *in vivo* therapeutic effect. Once cancer cells injected subcutaneously into the flank of NSG mice became palpable (approximately 0.1 cm^3^) mice were injected with ADCs. Graphs show therapeutic effect on tumor growth (left panel) and overall survival (right panel). **(A)** MIA PaCa-2 tumors treated at 4mg/kg/injection (left *P* < 0.001; right P < 0.0001) and 2mg/kg/injection (left *P* < 0.001; right *P* < 0.0001), **(B)** OV3331 patient-derived tumors treated at 3mg/kg/injection (left ns; right *P* < 0.05). ADCs were administered i.v. according to the treatment schedule indicated by the arrowheads. Data in **(A)** is from two independent experiments with *n* = 5-6 mice per group. Data in **(B)** is from two independent experiments with *n* = 4-5 mice per group.

To further evaluate the anti-tumor activity of PODO447-ADC, we next evaluated its efficacy against human tumors xenografted into immunocompromised NOD.Cg-*Prkdc^scid^ Il2rgtmWji/*SzJ (NSG) mice. These mice bear mutations in genes essential for the development of T, B and natural killer (NK) cells, rendering them unable to reject xenografts and are, thus, a widely used model to evaluate human tumor growth and anti-tumor therapeutics. Mice were injected subcutaneously with MIA PaCa-2 cells, which have previously been shown to grow rapidly in immunocompromised mice (46). Once tumors reached a size of 0.15 cm^3^, we began bi-weekly intravenous administration of PODO447-ADC at a dose of 4 mg/kg (**Figure 5A**). In order to monitor off-target toxicity, we used palivizumab-ADC as our control treatment. We found that four doses of PODO447-ADC led to complete tumor regression and significantly increased survival of these mice compared to control animals treated with palivizumab-ADC which exhibited only a minor and transient dip in tumor growth at the time of injection likely due to a low level of non-specific toxin uptake by tumors (**Figure 5A**, PODO447-ADC-treated median survival > 78 days *vs*. control-ADC-treated = 46 days, χ^2^ = 20.69). Similar results were obtained at a 2 mg/kg dose of PODO447-ADC, which also led to a dramatic increase in the length of survival of tumor bearing mice (PODO447-ADC-treated median survival > 56 days *vs*. control-ADC-treated = 43 days, χ^2^ = 22.61). In addition, to further evaluate the effects of ADC treatment on the PODO447 epitope and PODXL core protein expression *in vivo*, we isolated similar sized tumors from all treatment groups and evaluated them histologically for PODO447 or PODO83 (core protein) epitope expression. While virtually all cells of the palivizumab-ADC-treated tumors continued to express the PODO447 and PODO83 epitopes, in PODO447-ADC treated animals these epitopes were virtually absent in the 4 mg/kg treatment group and restricted to a core population of the cells in the center of tumors in the 2mg/kg cohort, possibly reflecting lower exposure of these core tumor cells to the ADC (**Supplementary Figure 5**). This intriguing result suggests that tumors that survive PODO447-ADC treatment do so by downregulating Podxl protein expression rather than simply altering the expression of the glycoepitope. Moreover, the much slower growth of PODO447-ADC treated tumors is consistent with our previous studies where shRNA-mediated dampening of Podxl expression in breast cancer cells suppressed tumor growth in mouse xenografts (16).

Because PODO447 recognizes the majority of high grade serous (HGS) ovarian carcinomas (32), we performed a similar experiment with the patient-derived HGS ovarian carcinoma line OV3331 (36). Again, bi-weekly intravenous injections of PODO447-ADC at 3 mg/kg dose, for a total of four doses, led to tumor regression and a striking increase in survival compared to control-ADC-treated group (**Figure 5B**, PODO447-ADC-treated median survival = 167 days *vs*. control-ADC-treated = 82 days, χ^2^ = 5.70). Importantly, there were no signs of adverse events or weight loss in any of the treatment groups. Thus, PODO447-ADC has a potent anti-tumor effect *in vivo* suggesting that PODO447-based therapeutics could prove beneficial for patients with Podxl-positive tumors.

### Enhanced PODO447-ADC Efficacy at Lower Doses in Animals With Functional B and NK Cells

**Figure 6:**
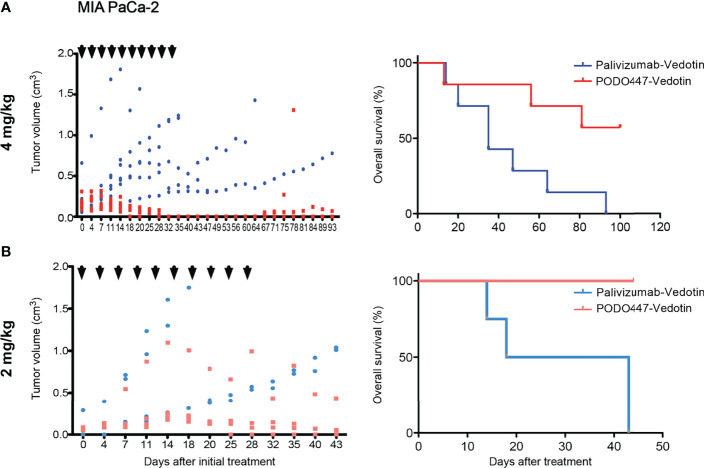
PODO447 therapeutic effect on pancreatic tumor bearing Nude mice with intact B and NK cell activity. PODO447- and palivizumab-ADC therapeutic effect on MIA PaCa-2 tumor growth (left panel) and overall survival (right panel) of tumor-bearing nude mice. Mice were treated with ADCs at **(A)** 4 mg/kg/injection (left *P* < 0.01; right *P* < 0.05) and **(B)** 2mg/kg/injection (left ns; right *P* < 0.001) following the treatment schedule indicated by the arrowheads. Data in **(A)** is from two independent experiments with *n* = 3-4 mice per group. Data in **(B)** is from one experiment with *n* = 4 mice per group.

Despite genetic differences, previous studies have noted that the efficacy of therapeutic Abs and ADCs and the level of off-target cytotoxicity varies widely depending on the degree of immunocompetence of the mouse model (47). Accordingly, we examined the effectiveness of PODO447-ADC in a second human tumor xenograft model. Specifically, we chose Nude mice, which bear a spontaneous chromosomal deletion of the *Foxn1* gene essential for the development of an appropriate thymic niche for T cell development. As a result, these mice are severely depleted of T cells but remain competent for the production of B and NK cells. Once again, we observed a strong therapeutic effect at similar doses of PODO447-ADC confirming our previous results. Treatment with PODO447-ADC at 4mg/kg/dose resulted in complete short-term tumor regression in all mice, and long-term regression in 4 out of 7 mice (no tumor relapse present at 100 days, **Figure 6A**, PODO447-ADC-treated median survival > 93 days *vs*. control-ADC-treated = 35 days, χ^2^ = 5.22). In contrast, control ADC at 4mg/kg had no significant effect on tumor burden, nor did it induce a transient dip in tumor growth. Additionally, we tested PODO447-ADC at 2 mg/kg/dose. While we observed a trend toward tumor regression in mice treated with PODO447-ADC compared to control, this did not reach statistical significance likely due to half of the control-treated mice reaching their humane endpoint very early (**Figure 6B**). Nevertheless, we found that treatment with 2 mg/kg of PODO447-ADC significantly increased OS and resulted in complete remission in 1 out of 4 mice as assessed at 125 days after initial treatment (**Figure 6B**, PODO447-ADC-treated median survival > 43 days *vs*. control-ADC-treated = 30.5 days, χ^2^ = 6.80). One intriguing explanation for this subtle difference between Nude and NSG mouse models is that Nude, but not NSG mice, have functional antibody secreting B cells and relatively normal levels of endogenous immunoglobulin in their serum which would be predicted to block non-specific antibody binding by tumor cells. Regardless, the data suggest that in two different xenograft models PODO447-ADC exhibits effective tumor killing at clinically relevant immunotherapeutic Ab doses.

## Discussion

Although novel immunotherapeutics (antibody- and cell-based) are providing an ever-increasing array of approaches to cancer treatments, several barriers continue to limit their broad-spectrum use, including failure to effectively target late-stage metastatic disease and safety concerns over targeting normal healthy tissues expressing reactive antigenic epitopes (toxicity). Accordingly, there has been a sustained effort to identify rare tumor-specific antigens expressed by a wide range of cancers that can effectively target molecules critical to late-stage disease progression. We find that the PODO447 Ab fulfills these key criteria.

In previous work, we and others have shown that the Podxl core protein is expressed by a wide variety of cancers and is consistently linked to poor outcome (10, 14–17, 32, 48). Although the new PODO447 Ab does not bind to all Podxl-expressing tumors, it does detect many including the majority of high grade serous ovarian tumors, highlighting its therapeutic potential (32). Here we developed this concept further and showed that, when used as the targeting arm of an ADC, PODO447 is highly effective at killing a range of tumor types including human glioblastoma, acute myelomonocytic leukemia, ovarian and pancreatic lines while sparing normal tissue as exemplified by the lack of cytotoxicity on HUVEC cells. Thus, PODO447-ADC could potentially be developed as a broad-spectrum immunotherapeutic to human cancers.

Our previous survey of normal human tissue revealed PODO447 to be exquisitely tumor-specific and lack reactivity with normal kidney podocytes or vascular endothelia, which abundantly express the Podxl core protein (32). This conclusion is further supported by the glycoepitope mapping data presented here, which shows that the PODO447 Ab selectively reacts with a cancer-associated core 1 O-glycostructure in the context of the Podxl polypeptide. *A priori*, this alleviates concerns regarding toxicity of this Ab on normal healthy tissue expressing Podxl and this is further supported by our data showing that PODO447-ADC fails to exhibit any substantial toxicity against normal HUVECs or against human cell lines where the Podxl gene has been inactivated, while simultaneously exhibiting dose-dependent killing of a broad spectrum of Podxl-positive tumor targets at clinically relevant concentrations *in vitro*. Finally, we provide key proof-of-concept data that PODO447-ADCs are effective *in vivo* and provide long-term tumor clearance in two immunodeficient animal models.

The finding that the residual tumors that survive PODO447-ADC treatment have downregulated not only the PODO447 epitope but also expression of the Podxl core protein is noteworthy and encouraging for several reason. First, it suggests that it may be difficult for PODO447+ human tumors to “escape” antibody treatment by simply altering the pattern of PODO447 glycosylation, a general concern for therapeutic strategies targeting these post-translational modifications. Secondly, it suggests that the enzymes that generate the PODO447 glycoepitope may be playing an important functional role for tumor behavior that is difficult to modify. Lastly, it is consistent with previous studies showing that ablation of Podxl in tumor lines cripples the ability of these cells to form tumors in xenografted mice, particularly during the metastatic phase of the disease (16, 27). Indeed, these reports highlight a critical role for Podxl in the subset of tumor cells responsible for tumorsphere formation *in vitro* and for colonization and establishment of tumors in specific niches *in vivo*, two key properties of tumor initiating cells (TIC). Thus, even in tumors where a minor subset of cells express Podxl, these data suggest that targeting this Podxl-expressing TIC population could target cells with the highest potential for the establishment of distal metastasis. Accordingly, targeting these cells could well have an outsized effect on disease progression. It will now be important to determine whether the PODO447 glycoepitope shows even greater specificity for tumor cells with TIC potential,

Notably, there is an ever-expanding array of antibody-based immunotherapeutic platforms gaining traction in treatment of neoplastic disease. Although our analyses here was limited to the use of PODO447 as an ADC, given its unusual tumor specificity, it holds equal promise for development as a radio-immunotherapeutic reagent for tumor imaging and killing, a bi-specific antibody for recruitment of T cells to tumors, or as a targeting arm for chimeric antigen receptor (CAR-T)-based therapeutic. Indeed, preliminary data suggest PODO447 is suitable for several of these additional applications (in preparation) and highlight the importance of antibodies with this rare specificity for future development.

## Data Availability Statement

The original contributions presented in the study are included in the article/**Supplementary Material**. Further inquiries can be directed to the corresponding authors.

## Ethics Statement

All animal experiments were carried out under approved University of British Columbia Animal Care committee protocols (ACC protocol #A16-0007 and A20-0042) based on the recommendations of the Canadian Council on Animal Care. This study was performed in consideration with the Tri-Council Policy Statement of Ethical Conduct for Research involving Humans (TCPS 2).

## Author Contributions

DCH designed and performed most experiments, analyzed and interpreted data, and was the primary author of this manuscript. MRH assisted with experimental design. YL assisted in flow cytometry and cytotoxicity assays. IMR provided the HEK-293-based glycan array data. PD created the SKOV3 and MIA PaCa-2 *PODXL-KO* cell lines and assisted in flow cytometry assays. JB provided the CFPAC-1 flow cytometry data. EMB created the CFPAC-1 sub-clones. IS provided the A172 ADCC data. AM provided and characterized the patient-derived ovarian carcinoma cell lines. HC, YN, and OB assisted with the glycan array experiments and analysis. CDR and KMM were key contributors to the experimental concept and design. All authors contributed to the article and approved the submitted version.

## Funding

This research was supported by the Canadian Institutes of Health Research (Grant Number: PJT-166180), the Canadian Cancer Research Institute (Grant number: 704344), the Danish National Research Foundation (Grant number: DNRF107), and European Union’s Horizon 2020 Research and Innovation Programme under the Marie Skłodowska-Curie grant agreement synBIOcarb (No. 814029).

## Conflict of Interest

The authors DC, MH, PD, IS, OB, KM, CR are inventors of a pending patent application on PODO447 and methods of using the same (US20180296673A1). MH, KM and CR are inventors of a pending patent application on PODO83 and methods of using the same (US20190367606A1). KM and CR possess an awarded patent on podocalyxin as a prognostic marker in cancer (US9309323B2).

The remaining authors declare that the research was conducted in the absence of any commercial or financial relationships that could be construed as a potential conflict of interest.

## Publisher’s Note

All claims expressed in this article are solely those of the authors and do not necessarily represent those of their affiliated organizations, or those of the publisher, the editors and the reviewers. Any product that may be evaluated in this article, or claim that may be made by its manufacturer, is not guaranteed or endorsed by the publisher.

## Supplementary Material

Supplementary Figure 1 Validation of HEK-293 isogenic library O-glycoform expression. Bar graphs summarizing flow cytometric binding of **(A)** anti-Tn (5F4), **(B)** anti-core 1 (3C9), and **(C)** anti-STn (TKH2) antibodies; and **(D)** anti-pan-specific sialoglycan (Pan-Lectenz), **(E)** anti-GalNAc (*Wisteria floribunda*, WFA), and **(F)** anti-Gal, -GalNAc and -Lactose (*Erythrina cristagalli* lectin, ECL) binding lectins to HEK-293 WT and isogenic cells expressing dST (HEK-293 KO GCNT1), ST (HEK-293 KO GCNT1/ST6GALNAC2/3/4), T (HEK-293 KO GCNT1/ST6GALNAC2/3/4/ST3GAL1/2), Tn (HEK-293 KO C1GALT1), STn (HEK-293 KO COSMC/KI ST6GALNAC1), Core 3 (HEK-293 KO COSMC/KI B3GNT6), and lacking expression of LacDiNAc (ΔLacDiNAc, HEK-293 KO B4GALT1/2/3/4).

Supplementary Figure 2 PODO447 recognizes a T-antigen epitope on the Podxl core protein. Flow cytometric binding profiles of CFPAC-1 parental, PODO447-positive and PODO447-negative subclones co-stained with anti-core 1 PNA lectin and either PODO83 or PODO447.

Supplementary Figure 3 Validation of linker stability and biotin/MMAE conjugation. **(A)** Cytotoxic effect of PODO447- and palivizumab-Vedotin on neutrophil differentiation represented by the percentage of viable CD66b+ cells as assessed by flow cytometry after 144 h of continuous exposure. Comparison by flow cytometry of **(B)** unconjugated or biotinylated PODO447 binding profile to SKOV3 parental cells; and **(C)** unconjugated or MMAE-conjugated PODO447 binding profile to SKOV3 Podxl KO cells re-expressing the full-length Podxl protein.

Supplementary Figure 4 PODO83 and PODO447 binding profile of tumor and normal cells. Flow cytometric PODO83 (blue lines) and PODO447 (red lines) binding profiles of Podxl-positive normal (HUVEC), glioblastoma (A-172), medulloblastoma (ONS-76), acute monocytic leukemia (THP-1), patient tumor-derived high grade serous ovarian carcinoma (OV3331, TOV3133D), ovarian (SKOV3) and pancreatic (MIA PaCa-2) cells lines.

Supplementary Figure 5 PODO83 and PODO447 staining of ADC-treated tumors. Tumor sections from PODO447- or palivizumab-Vedotin treated animals at 2 and 4 mg/kg doses stained with either PODO447, PODO83 (core protein), palivizumab control or rabbit IgG control antibody.

## Abbreviations

Ab(s), antibodies; ADC, antibody drug conjugate; AML, acute myeloid leukemia; HGS, high grade serous; KI, knock-in; KO, knockout; NK, natural killer cells; NSG, NOD.Cg-Prkdcscid Il2rgtmWji/SzJ; Podxl, podocalyxin; RSV, respiratory syncytial virus; T, T-antigen.
