# Supplementary figures and images for: Targeting a Tumor-Specific Epitope on Podocalyxin Increases Survival in Human Tumor Preclinical Models
Source: Front Oncol. 2022 May 4;12:856424. doi: 10.3389/fonc.2022.856424 (PMC9115113; doi:10.3389/fonc.2022.856424)

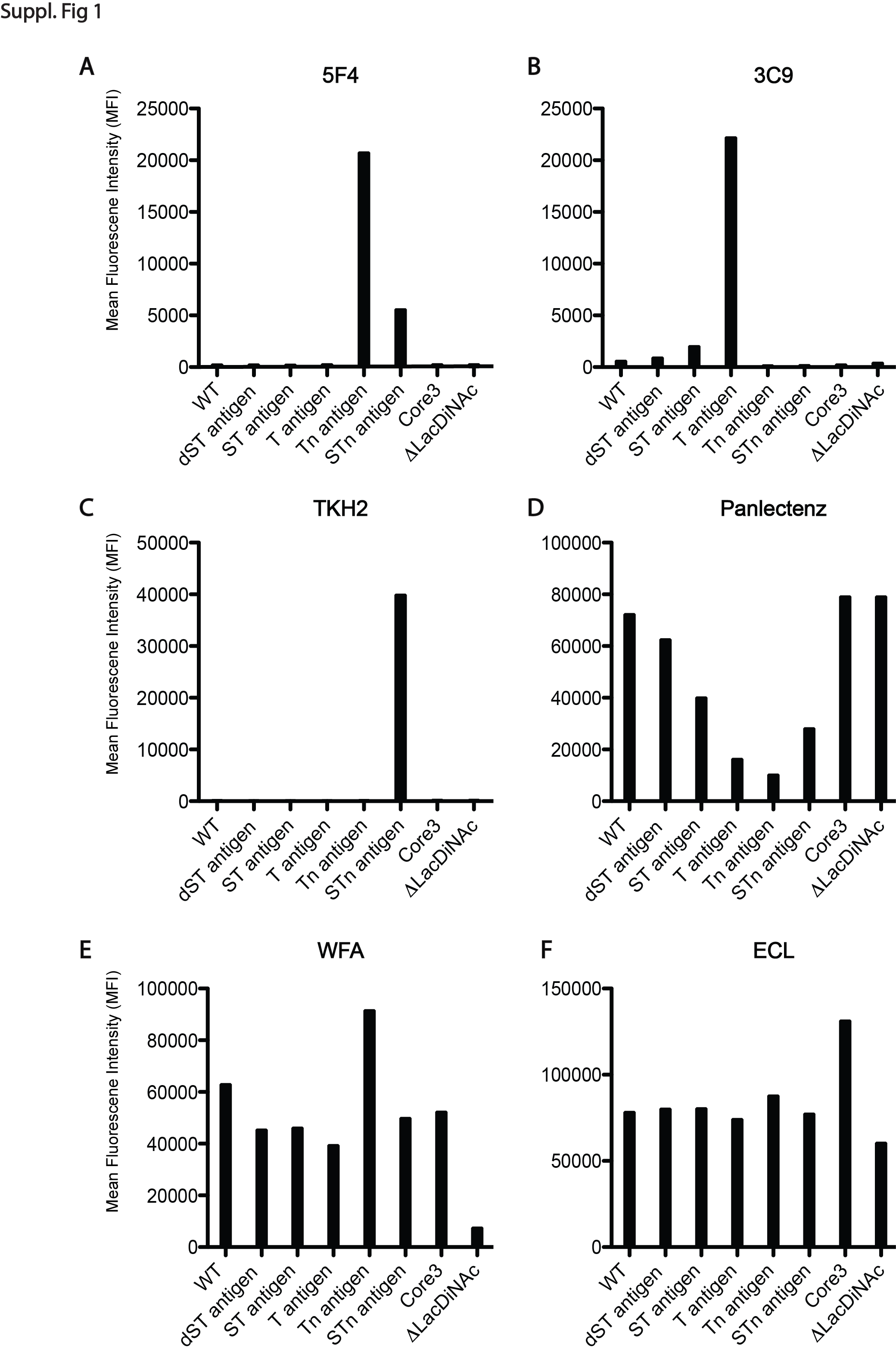

Supplement: Supplementary Figure 1 — Validation of HEK-293 isogenic library O-glycoform expression. Bar graphs summarizing flow cytometric binding of (A) anti-Tn (5F4), (B) anti-core 1 (3C9), and (C) anti-STn (TKH2) antibodies; and (D) anti-pan-specific sialoglycan (Pan-Lectenz), (E) anti-GalNAc (Wisteria floribunda, WFA), and (F) anti-Gal, -GalNAc and -Lactose (Erythrina cristagalli lectin, ECL) binding lectins to HEK-293 WT and isogenic cells expressing dST (HEK-293 KO GCNT1), ST (HEK-293 KO GCNT1/ST6GALNAC2/3/4), T (HEK-293 KO GCNT1/ST6GALNAC2/3/4/ST3GAL1/2), Tn (HEK-293 KO C1GALT1), STn (HEK-293 KO COSMC/KI ST6GALNAC1), Core 3 (HEK-293 KO COSMC/KI B3GNT6), and lacking expression of LacDiNAc (ΔLacDiNAc, HEK-293 KO B4GALT1/2/3/4). [file Image_1.tif]

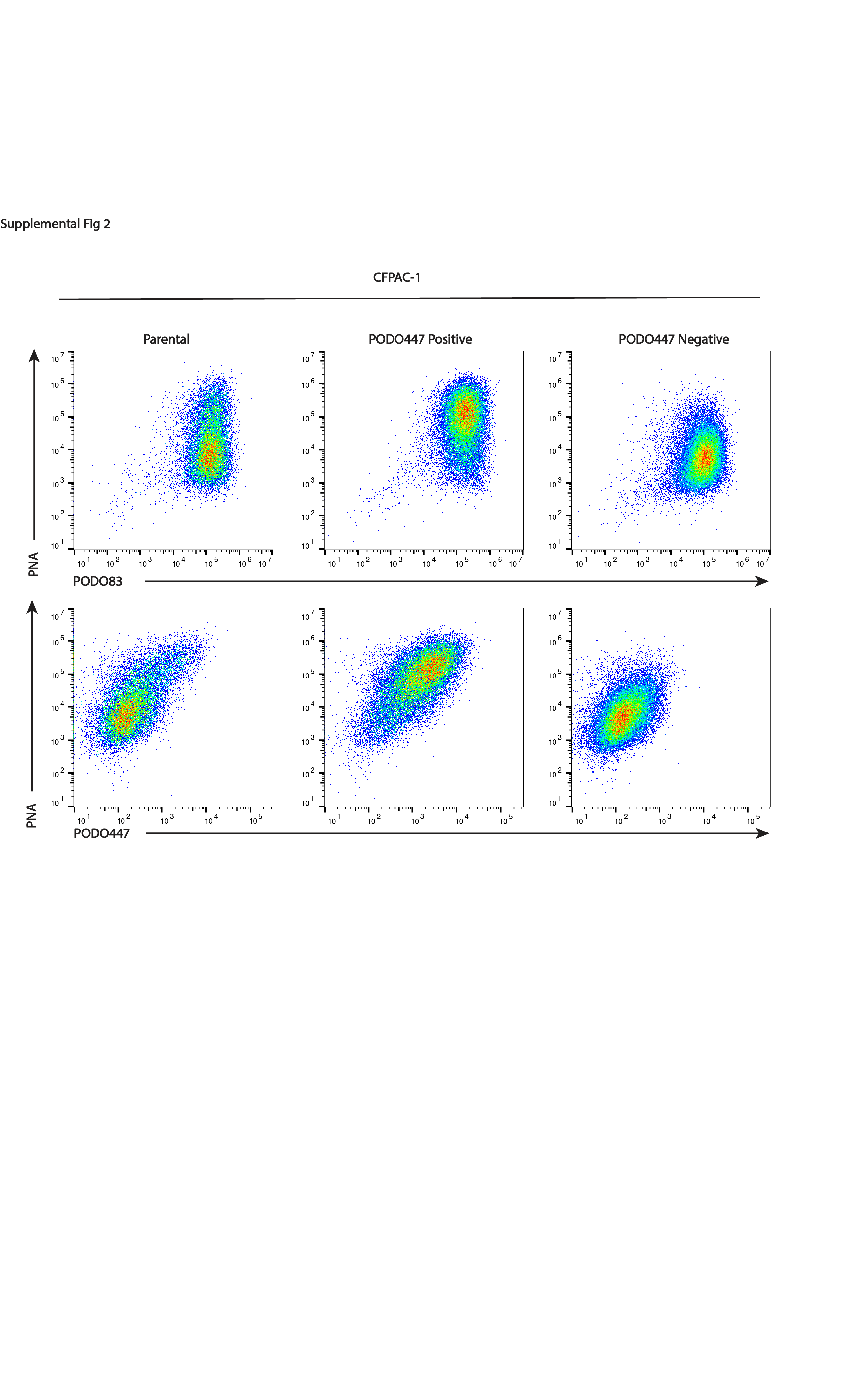

Supplement: Supplementary Figure 2 — PODO447 recognizes a T-antigen epitope on the Podxl core protein. Flow cytometric binding profiles of CFPAC-1 parental, PODO447-positive and PODO447-negative subclones co-stained with anti-core 1 PNA lectin and either PODO83 or PODO447. [file Image_2.tif]

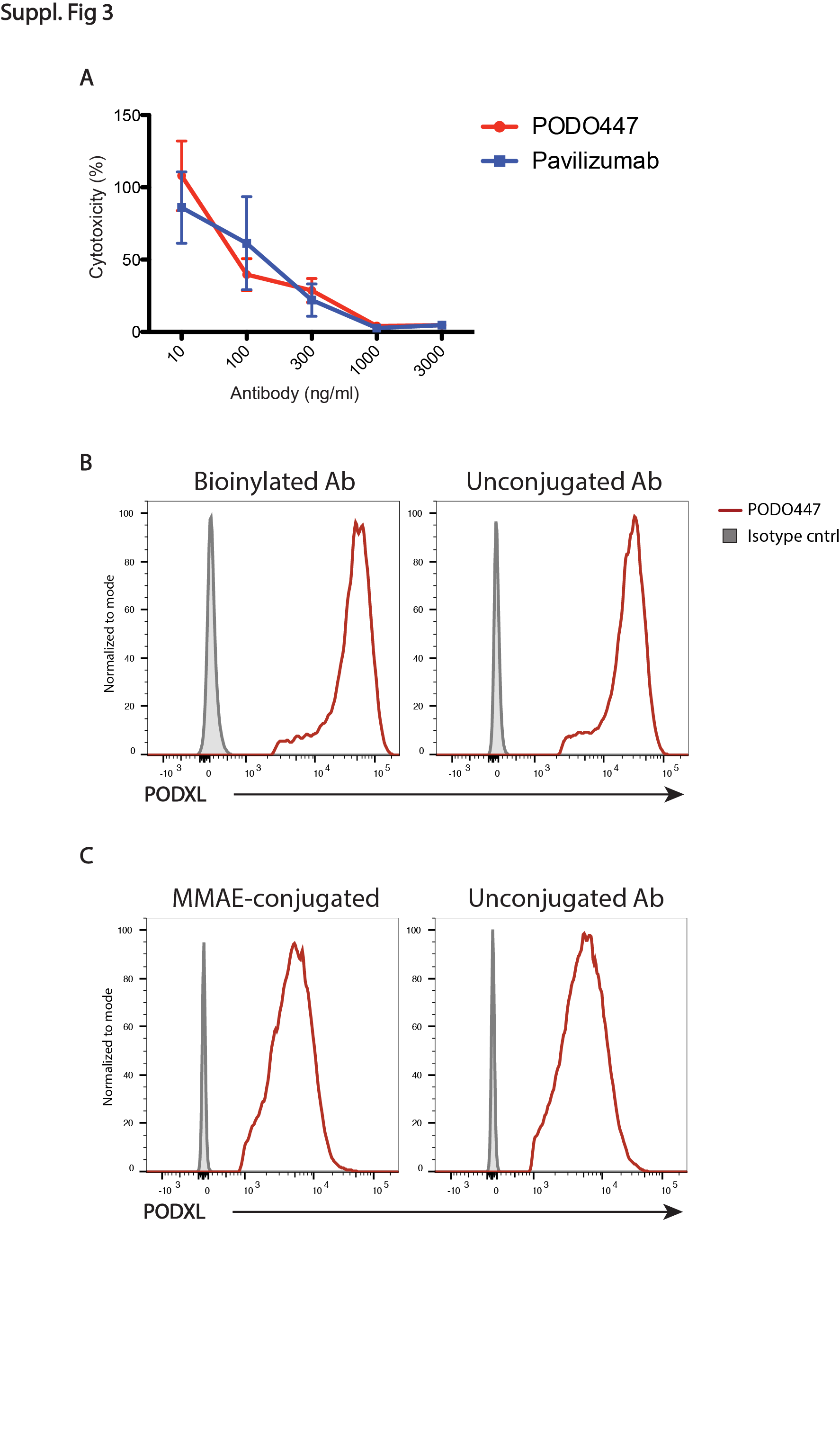

Supplement: Supplementary Figure 3 — Validation of linker stability and biotin/MMAE conjugation. (A) Cytotoxic effect of PODO447- and palivizumab-Vedotin on neutrophil differentiation represented by the percentage of viable CD66b+ cells as assessed by flow cytometry after 144 h of continuous exposure. Comparison by flow cytometry of (B) unconjugated or biotinylated PODO447 binding profile to SKOV3 parental cells; and (C) unconjugated or MMAE-conjugated PODO447 binding profile to SKOV3 Podxl KO cells re-expressing the full-length Podxl protein. [file Image_3.tif]

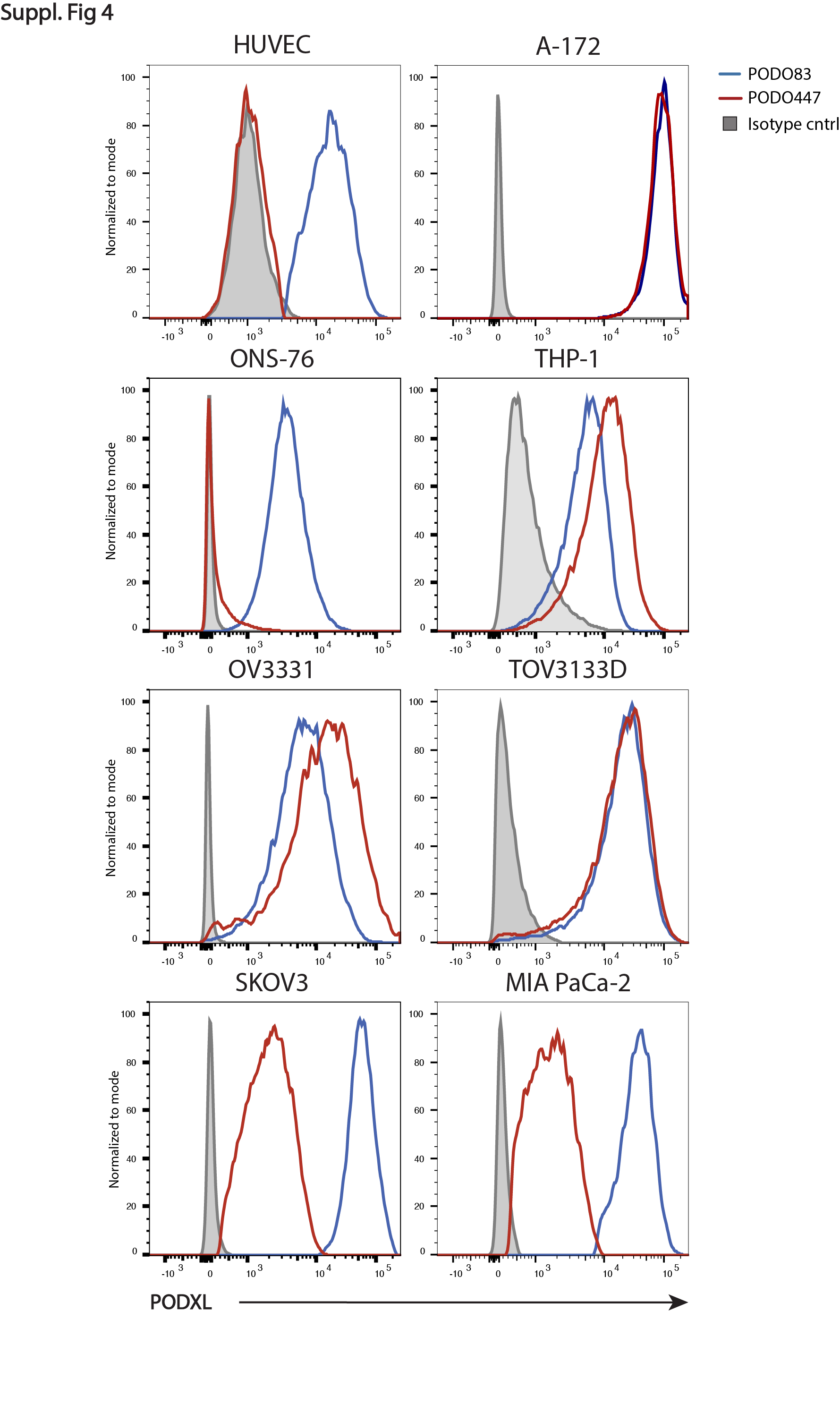

Supplement: Supplementary Figure 4 — PODO83 and PODO447 binding profile of tumor and normal cells. Flow cytometric PODO83 (blue lines) and PODO447 (red lines) binding profiles of Podxl-positive normal (HUVEC), glioblastoma (A-172), medulloblastoma (ONS-76), acute monocytic leukemia (THP-1), patient tumor-derived high grade serous ovarian carcinoma (OV3331, TOV3133D), ovarian (SKOV3) and pancreatic (MIA PaCa-2) cells lines. [file Image_4.tif]

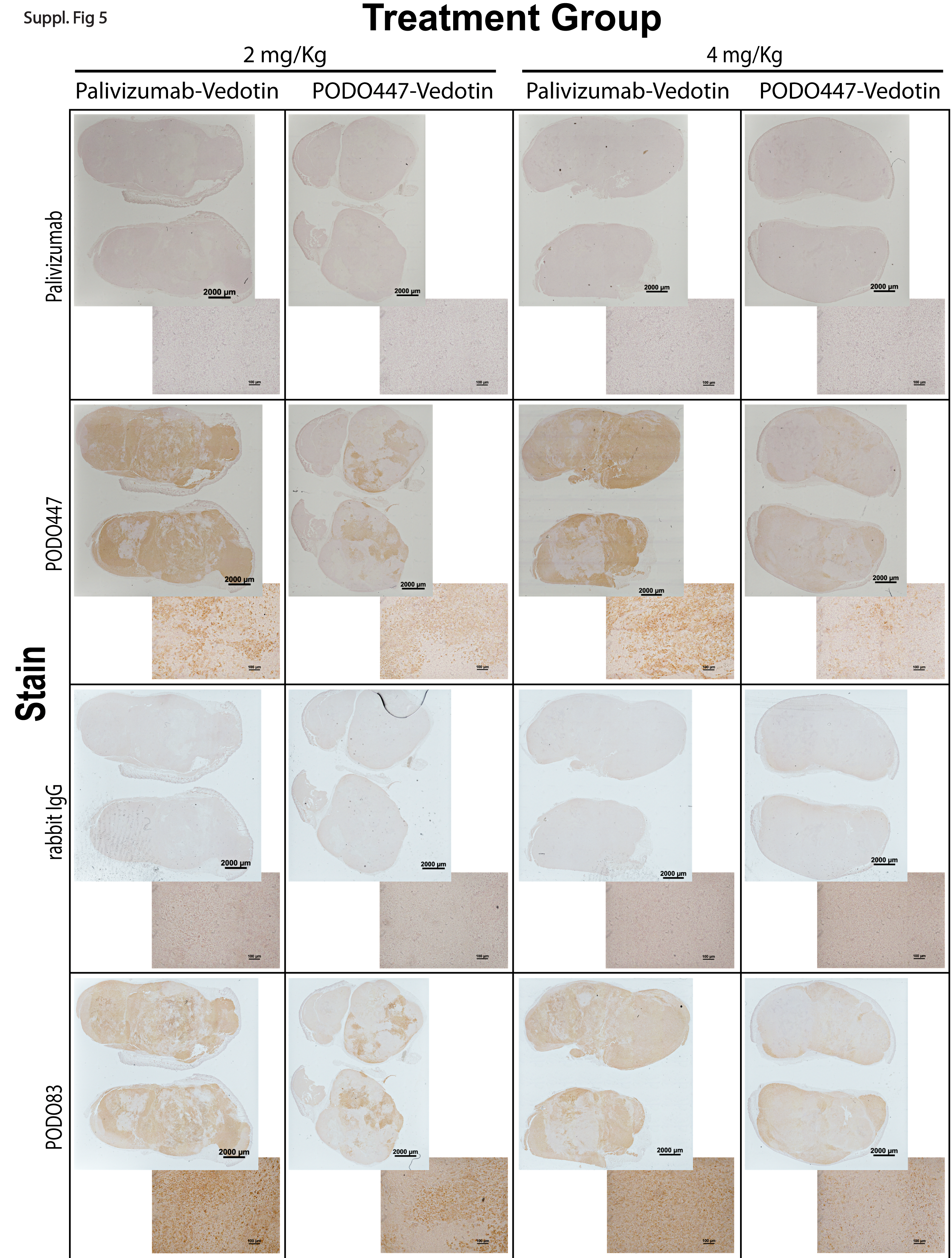

Supplement: Supplementary Figure 5 — PODO83 and PODO447 staining of ADC-treated tumors. Tumor sections from PODO447- or palivizumab-Vedotin treated animals at 2 and 4 mg/kg doses stained with either PODO447, PODO83 (core protein), palivizumab control or rabbit IgG control antibody. [file Image_5.tif]
